# Total resection of an infected aortic arch aneurysm caused by *Mycobacterium avium*: a case report

**DOI:** 10.1186/s13019-019-0972-1

**Published:** 2019-08-19

**Authors:** Kei Yagami, Takashi Fujita, Shinichi Ishida, Masato Mutsuga

**Affiliations:** 1 Gifu Prefectural Tajimi Hospital Cardiac Surgery, 161-5, Maebata-cho, Tajimi-City, Gifu 507-8532 Japan; 2 Department of Cardiac Surgery, Nagoya University Graduate School of Medicine, Nagoya,, Japan

**Keywords:** Nontuberculous mycobacteria, *Mycobacterium avium*, Infected aneurysm, Aortic arch aneurysm, Total arch replacement, Rifampicin-bonded gelatin-sealed woven Dacron graft

## Abstract

**Background:**

Infected aortic arch aneurysms caused by *Mycobacterium avium* are rare in immunocompetent individuals. Promptly recognizing these aneurysms is important because delays in treatment result in aneurysm rupture and a high fatality rate. Although *Salmonella* species, *Streptococcus* species, *Staphylococcus aureus*, and *S*. *epidermis* are commonly found in immunocompetent individuals, to our knowledge, infected aortic arch aneurysms caused by *M. avium* have not yet been reported.

**Case presentation:**

We report the case of a 63-year old immunocompetent man who underwent total arch replacement following infection by the nontuberculous mycobacteria *M. avium*. The procedure involved total aneurysmal resection and arch replacement with a rifampicin-bonded gelatin-sealed woven Dacron graft. He was discharged without complications and remained asymptomatic after 30 months.

**Conclusion:**

In this brief report, we outline and discuss the rare successful case of total arch replacement using total aneurysmal resection and rifampicin-bonded gelatin-sealed woven Dacron graft for an infected aortic arch aneurysm resulting from *M. avium* in an immunocompetent patient.

## Background

Infected aortic arch aneurysms caused by *Mycobacterium avium* are very rare in immunocompetent individuals [1, 2]. Promptly recognizing these aneurysms is important because delays in treatment results in aneurysm rupture and a high fatality rate. Although *Salmonella species, Streptococcus* species, *Staphylococcus aureus,* and *S*. *epidermis* are commonly found in immunocompetent individuals, to our knowledge, infected aortic arch aneurysms caused by *M. avium* and their successful repair have not yet been reported. Here, we report a rare case of total arch replacement using a rifampicin-bonded gelatin-sealed woven Dacron graft for an infected aortic arch aneurysm resulting from *M. avium*.

## Case presentation

The patient was a 63-year-old man who was diagnosed with malignant lymphoma in 2013 and underwent 2 years of radiotherapy and chemotherapy, after which he was deemed to be in complete remission. During the course of chemotherapy, the patient’s white blood cell count reduced to 200/μl, and he was in an immunocompromised state, but at the end of the chemotherapy, the count recovered to normal, and the patient lived a normal life without any medication. In 2016, mediastinal lymphadenopathy was noted on a follow-up chest computed tomography (CT), and his history revealed a dry cough for several months. Therefore, bronchoscopic lymph node biopsy, sputum culture, and blood culture were performed. Although the blood culture was negative, sputum culture grew *M. avium*, and he was diagnosed with nontuberculous mycobacterial infection and started on a course of rifampicin 570 mg/day, ethambutol 750 mg/day, and clarithromycin 800 mg/day.

**Fig. 1:**
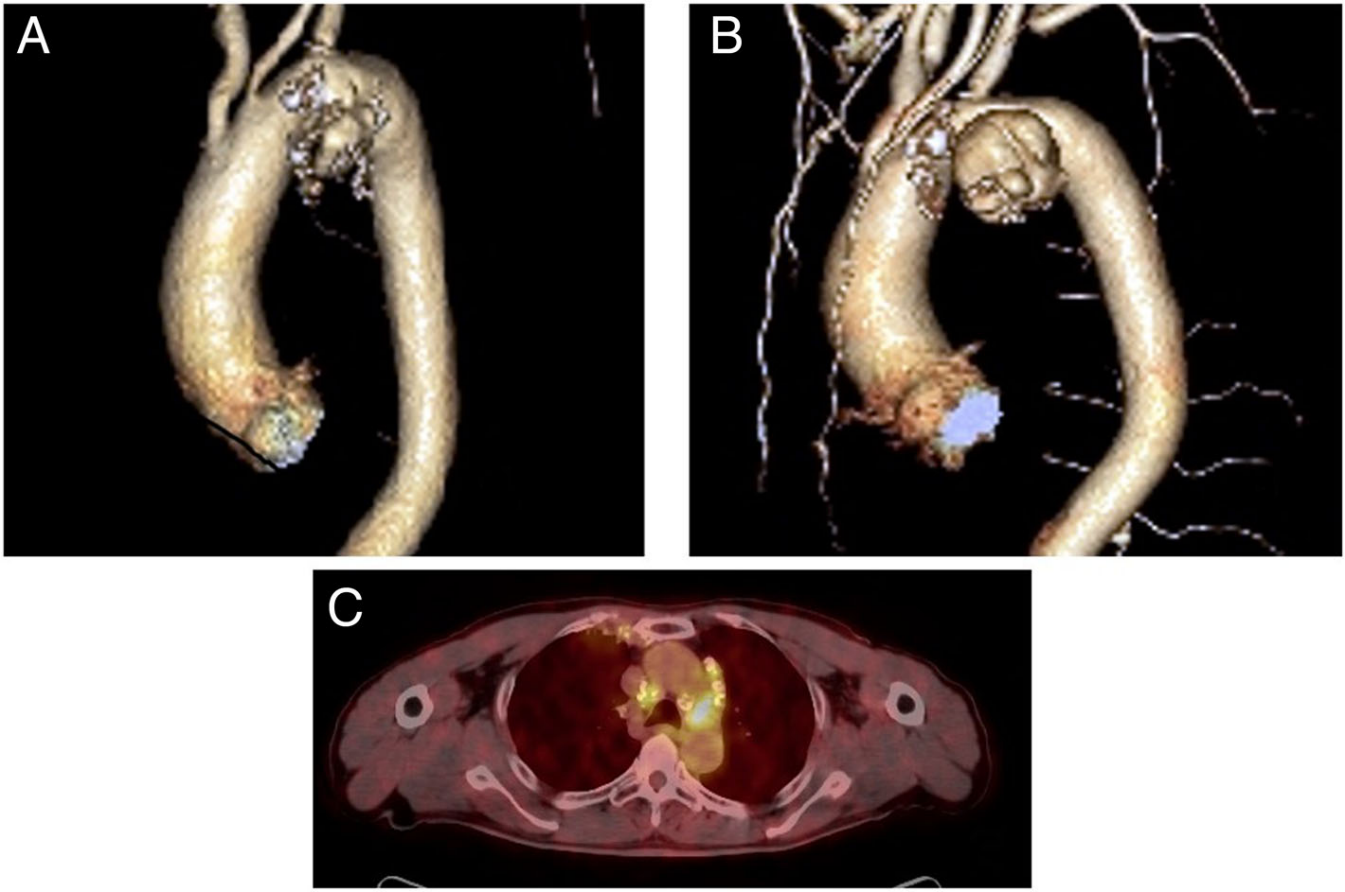
**a** Computed tomography (CT) of the chest revealed a new saccular aneurysm of the aortic arch. **b** After 1 month, follow-up CT showed a rapidly expanding saccular aneurysm of the aortic arch. **c** F-18 fluorodeoxyglucose positron emission tomography with computed tomography showing intense fluorodeoxyglucose avidity in soft tissue involving the aneurismal segment of the aortic arch

After 1 month, he developed low-grade fever and chest pain, and a chest CT revealed a new saccular aneurysm of the aortic arch measuring 41 × 49 mm (Fig. 1a). He was continued on antibiotic treatment and closely followed; however, after 1 month a follow-up chest CT revealed that the saccular aneurysm had expanded to 43 × 62 mm (Fig. 1b), suggesting false aneurysm formation. 18F-fluorodeoxyglucose (FDG) positron emission tomography with CT (PET/CT) was also performed for suspected infective aneurysm, and this revealed FDG uptake in the aneurysm (Fig. 1c). Therefore, the patient was diagnosed with an infected aortic arch aneurysm due to *M. avium*.

Early surgical repair was deemed necessary because of the rapid expansion of the aneurysm. The options available were total arch replacement or thoracic endovascular aortic repair (TEVAR) with one or two debranching arch vessels as options. However, we opted to proceed with total arch replacement using a rifampicin-bonded gelatin-sealed woven Dacron graft because we strongly suspected an infective etiology. At surgery, median sternotomy was performed, and cardiopulmonary bypass was established via the femoral artery and the superior and inferior vena cava cannulas. Systemic anticoagulation was initiated with 300 units/kg heparin. Under hypothermic circulatory arrest at a pharyngeal temperature of 23 °C and a rectal temperature of 25 °C, the aortic arch was opened, and balloon-tipped selective cerebral perfusion cannulas were inserted. Balloon-occluded distal anastomosis was then performed under femoral perfusion of the lower body. The aneurysm was extensively debrided, and the resected section was sent for culture. Finally, the aortic arch was replaced with a rifampicin-bonded gelatin-shield 30-mm 4 branch woven Dacron graft (J Graft Shield Neo; Japan Lifeline Co., Ltd., Japan). Because of the presence of infected aneurysms near the origin of left subclavian artery, we could not perform in situ reconstruction of the artery and performed only extraanatomical reconstruction. The origin of left subclavian artery was closed, and the reconstruction was peripherally performed.

**Fig. 2:**
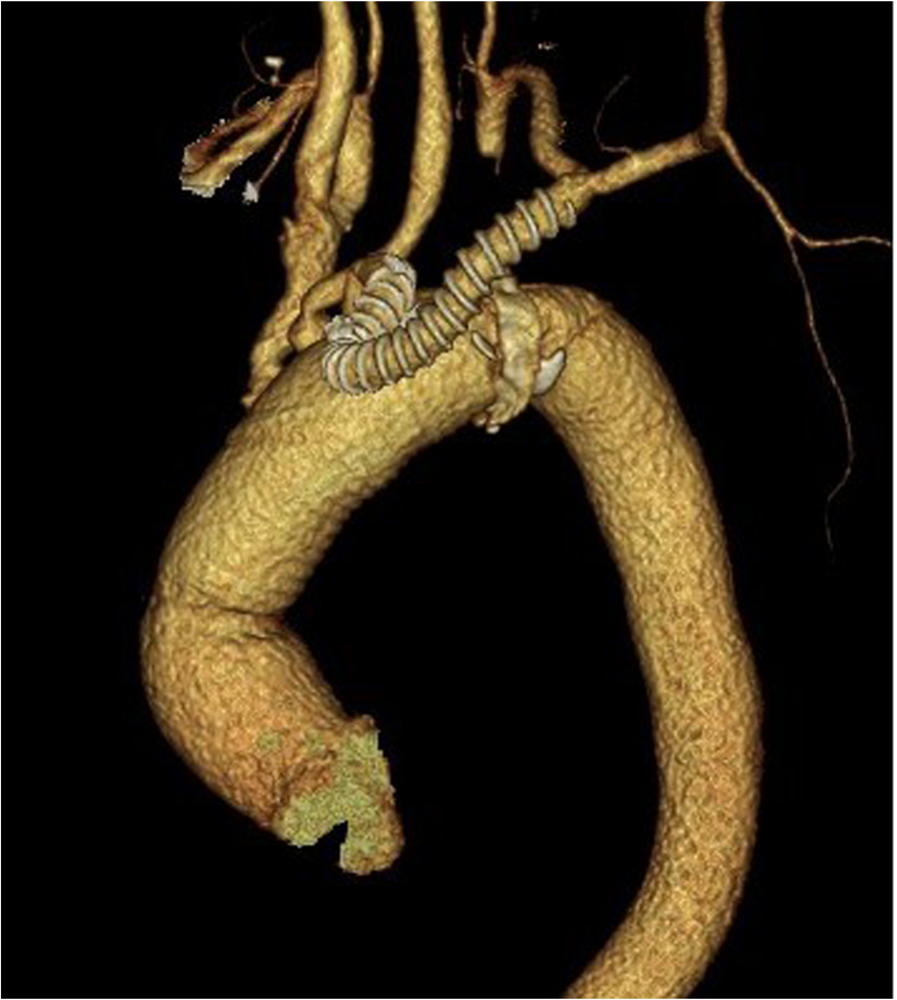
Postoperative computed tomography (CT) showed no aneurysm of the aortic arch and complete resection of the lesion

Postoperatively, no major complications were observed, and CT at the 14th postoperative day confirmed complete resection of the aneurysm (Fig. 2). *M. avium* was isolated from the resected tissue. The patient was discharged on the 15th postoperative day without incident and continued on the course of rifampicin 570 mg/day, ethambutol 750 mg/day, and clarithromycin 800 mg/day. He has received oral antibiotic therapy as an outpatient for 30 months with no symptoms, and we plan to continue them for several more months.

## Discussion and conclusions

Nontuberculous mycobacteria are rarely pathogenic in immunocompetent individuals, but they are important pathogens in immunocompromised individuals [1, 2]. In immunocompetent individuals, aneurysm infections are typically with *Salmonella* species, *Staphylococcus aureus, S. epidermis*, or *Streptococcus* species. In our case, the patient had previously undergone radiotherapy and chemotherapy for malignant lymphoma, so will have developed a temporary immunocompromised status that could have accounted for his rare presentation.

CT is the standard tool for imaging suspected infected aneurysms. It provides a detailed assessment of the aneurysm and can offer morphological and anatomical images in high resolution. Periaortic fat, contrast enhancement, or fluid collections are clues to infection, but such features are not always prominent and may be missed in low-grade infection. In suspected cases that cannot be diagnosed by CT, diagnosis can be confirmed by 18F-FDG PET/CT [3], as in the current report.

TEVAR has been reported to have good results in the treatment of infected aneurysms. However, the risks of repeat infection are high after repair, not least because it requires that the stent graft be implanted in an infected aorta, and the procedure can be used for bridging to surgical repair [4]. Consequently, several permanent approaches have been reported to treat infected aortic aneurysms that use rifampicin-bonded grafts, homografts, or omental wrapping. Raman et al. reported an excellent outcome when using a rifampicin-bonded in situ graft for an infected abdominal aortic aneurysm due to *M. avium* in an immunocompromised patient [1]. Their patient was discharged on the seventh postoperative day and remained asymptomatic after 2 years of follow-up. In contrast, Tsilimparis et al. reported the use of a homograft for an infected abdominal aortic aneurysm caused by *M. avium* in an immunocompromised patient [2]. However, their patient developed multiorgan failure and had care withdrawn on the second postoperative day. Uchida et al. reported excellent results with the use of rifampicin-bonded and omental pedicle grafts in 23 cases of infected thoracic, thoracoabdominal, or abdominal aortic aneurysms [5], but their procedure was for mycotic rather than nontuberculous mycobacterial aneurysms. Some reports have reported good results with prosthesis made from bovine pericardium in the setting of infection [6].

It is difficult to prepare homografts at the time of surgery in Japan. It was also considered difficult to use the omentum in the present case because the patient had previously undergone abdominal surgery, and we considered that the aortic arch replacement using the bovine pericardium was morphologically difficult. Therefore, we considered open surgical repair with aneurysmal resection and rifampicin-bonded grafting as better realistic therapy. At present, total resection of the infected aneurysmal wall and replacement with a rifampicin-bonded graft, with or without an omental pedicle, may be the optimal therapy. *M. avium* infection of an aneurysm in the thoracic aortic is rare in immunocompetent patients. We report the diagnosis in a patient who was successfully treated with a rifampicin-bonded gelatin-shield woven Dacron graft. Meticulous follow-up is mandatory for these cases, and therapeutic experience needs to be accumulated.

## Data Availability

Not applicable.

## Abbreviations

CT: Computed tomography
FDG: Fluorodeoxyglucose
PET/CT: Positron emission tomography with computed tomography
TEVAR: Thoracic endovascular aortic repair
